# Annotations

**Published:** 1902-05-10

**Authors:** 


### ANNOTATIONS.

### New Milk.

It has long been a question what is meant by the word milk, and we gather from a decision given in the Court of King's Bench Division, last week, that no amount of proof that any sample was sold to the customer in precisely the condition in which it was milked from the cow, will of necessity be accepted as proving its genuineness. It is only right to say that Mr. Justice Darling disagreed. He said that the purchaser asked for new milk, and he got milk exactly as it came from the cow. What he got was undoubtedly new, and the question was, was it milk? If it was not milk what was it? Some cows gave richer milk than others, and there being no standard the appellant supplied what he was perfectly entitled to describe as milk. He went further, indeed, and pointed out that it would be a principle capable of dangerous application to say that a man could be convicted where without any element of fraud he sold a natural product in the state in which it was produced, illustrating his contention by the case of apples. If apples, sold straight off the tree, be shown not to contain a due quantity of malic acid are they therefore not apples? And what are they? Nevertheless, he was overruled, owing to the fact that evidence had been given that an abnormal time had been allowed to elapse between the milkings, which, it was stated, would cause the secretion of an abnormally poor milk. The Lord Chief Justice said that "The milk was deficient in 30 per cent. of the fat proper to genuine milk. If there had been evidence that the milk came direct from the cow, and no evidence of an abnormal condition of things, it would have been wrong to convict. But in this case it was found that the condition of the milk was due to the way in which the cows had been milked, *i.e.*, there had been an abnormal interval of 16 hours." He went on to say that although milk might not come up to the standard established by the Board of Agriculture, it could still be proved by the defendant that it was genuine milk. "But if it turned out that the article was produced by an abnormal condition of things, whether from disease or from abnormal treatment, there was evidence on which the justices might find that the article was not of the nature, substance, and quality demanded." This decision has many important bearings.

### Boiled Water for the Troops.

At last our soldiers are to be provided with boiled water. The water will first of all be clarified by a kind of rough filtration through charcoal containing a certain amount of potassium permanganate and will then be "sterilised" either by filtration or by heat, after which it will be distributed to the troops by means of water-carts reserved for "safe" water only. So far as the method of sterilisation by boiling is concerned, the War Office has adopted a form of apparatus which is very economical of heat, being so arranged that the incoming water absorbs the heat from that which has been through the boiler, and this so effectually that although the water is raised to boiling temperature it flows out only 4½° Fahr. hotter than it entered the apparatus. By this process active pathogenic organisms are destroyed, although a few of such as happen to be present in the form of spores may escape. In the portable form of apparatus the water does not flow out quite so cool as in the larger form, but this is regarded as fully compensated for by its lesser weight, the field apparatus weighing only 90 lb., and being capable of yielding 125 gallons at an expenditure of one quart of petroleum. Having got the apparatus, the question now is, What will the British Army do with it? This depends, not upon the Medical Department, but upon the extent to which commanding officers can be brought to see the necessity of insisting upon boiled water alone being used for drinking purposes.

### The Prevention of Tuberculosis.

The last utterance on the subject of tuberculosis is that of Professor Behring, who has satisfied himself, notwithstanding the statements of Professor Koch, that the differences observed between tubercle bacilli from human beings and those from cattle are merely occasioned by the adaptation of the bacillus to the conditions of life in the respective organisms. We are further told that he has rendered young cattle immune against virulent tubercle by means of previous inoculation with living tubercle bacilli of inferior virulence, and that he thinks this treatment will prove of great value in combating the disease. Let us hope that medical fashion will not be tempted to follow this lead. Tuberculosis is immediately due, as we all admit, to the growth of a bacillus, but the disease, as it exists in man, is the definite product of certain insanitary modes of life, and although it is quite conceivable that by some form of inoculation with bacilli of inferior virulence we might obtain protection from this particular penalty of our sanitary sins, any such proceeding would merely tend to delay that all-round improvement of the conditions of life which is the real weapon by which tuberculosis has to be fought. A vaccination which should prove successful against the more prominent ill effects of close and dirty living could but tend to lower still further the standard of comfort which is permitted in civilised communities, a consummation by no means to be desired.

### Small-pox and Country Holidays.

Certain good people are in the habit of sending a large number of poor London children into the country every summer for a holiday. Unfortunately, just at present, the sanitary authorities of country districts do not look very kindly upon such an importation of children of the slums, fearing that they may bring small-pox with them, and some anxiety has been expressed lest this year these thrice-blessed country holidays should fall through. If, however, care be taken that no children be sent except those who can produce good evidence of efficient vaccination, or, in cases over 10 years of age, of having been re-vaccinated, the danger of their introducing small-pox should be very slight, and far less than that of their taking with them the seeds of diphtheria and other maladies which are far more difficult to control. The offer of fifty thousand country holidays a year, to be distributed among vaccinated children only, ought to prove a mighty engine for good.

